# Collagen and fibronectin surface modification of nanoporous anodic alumina and macroporous silicon for endothelial cell cultures

**DOI:** 10.1186/s13036-018-0111-x

**Published:** 2018-10-01

**Authors:** P. Formentín, Ú. Catalán, L. Pol, S. Fernández-Castillejo, R. Solà, L. F. Marsal

**Affiliations:** 1 Departament d’Enginyeria Electrònica, Elèctrica i Automàtica, Universitat Rovira i Virgili, Països Catalans 26, 43007 Tarragona, Spain; 2 Functional Nutrition, Oxidation, and Cardiovascular Diseases Group (NFOC-Salut), Hospital Universitari Sant Joan (HUSJR), Institut d’Investigació Sanitaria Pere Virgili (IISPV), Faculty of Medicine and Health Sciences, Universitat Rovira i Virgili, Sant Llorenç, 21, 43201 Reus, Spain

**Keywords:** Macroporous silicon, Nanoporous anodic alumina, Endothelial cells, Collagen adhesion, morphology and proliferation, Fibronectin, Surface properties

## Abstract

**Background:**

The ability to direct the cellular response by means of biomaterial surface topography is important for biomedical applications. Substrate surface topography has been shown to be an effective cue for the regulation of cellular response. Here, the response of human aortic endothelial cells to nanoporous anodic alumina and macroporous silicon with collagen and fibronectin functionalization has been studied.

**Methods:**

Confocal microscopy and scanning electron microscopy were employed to analyse the effects of the material and the porosity on the adhesion, morphology, and proliferation of the cells. Cell spreading and filopodia formation on macro- and nanoporous material was characterized by atomic force microscopy. We have also studied the influence of the protein on the adhesion.

**Results:**

It was obtained the best results when the material is functionalized with fibronectin, regarding cells adhesion, morphology, and proliferation.

**Conclusion:**

These results permit to obtain chemical modified 3D structures for several biotechnology applications such as tissue engineering, organ-on-chip or regenerative medicine.

## Background

Porous materials are studied in a variety of systems for drug delivery and tissue engineering, which is an interdisciplinary field that applies the principles of biology and engineering to the development of functional substitutes that restore or improve the function of the damaged tissue [1–3]. Cellular response is affected by the environment of the substrate on which the cells are cultured, which in turn influences cell-substrate interactions and cell adhesion, morphology, migration, or differentiation [4–8]. Topographic and chemical features of cell substrates are appropriate for the cell-material interaction control [9–11]. Reactions of cells to topography are different in the nanometer and micrometer range [12–18]. Nanoporous anodic alumina (NAA) and porous silicon (PSi) are considered structural biomaterials for medical applications and can be used as substrates for cells culture due to its characteristics [19–30]. Silicon dioxide is nontoxic, biodegradable and dissolves into nontoxic silicic acid. Its surface stability and solvent compatibility are features to its application in biotechnology and biomedicine. Nanoporous anodic alumina is a type of ordered nanomaterial with regular pore size. It is optically transparent, chemically stable, bioinert and biocompatible. These properties are beneficial for applications of NAA in medicine.

The macro- or nanostructures on these materials cause effects on cell behaviors, which could be manipulated via tuning the biophysical properties of the structures. Nanoporous anodic alumina is a self-organized material with nanopore arrays. The porous structure can be altered by varying anodization processing parameters and the resulting porous shapes can be tailored with specific pore diameters [31–33]. PSi is fabricated by means of anodization of monocrystalline wafers and degrades into orthosilicic acid when in contact with an aqueous environment, which is the bioavailable form of silicon [34, 35]. The structural tuneability of the PSi allows a range of pore sizes from microporous to macroporous.

An effective way to control cell adhesion from a porous material is to improve cell-surface interaction by surface chemical functionalization with proteins since it is well known that cells grow and attach better on a functionalized surface than on a non-functionalized surface [19, 36–39]. Several activated surfaces using biological components such as proteins have been introduced to improve the substrate properties such as biocompatibility and hydrophilicity. Among the covalent-binding strategies, material surfaces chemically modified with amino silanes and homobifunctional aldehydes, such as glutaraldehyde (GTA), have shown efficiency in immobilizing proteins and antibodies [40, 41]. The efficiency of 3-aminopropyltrietoxysilane (APTES) + GTA-modified porous surfaces in immobilizing extracellular matrix proteins, such as collagen (Col) or fibronectin (Fn) and, the biocompatibility of these modified surfaces for the adhesion and proliferation of human aortic endothelial cells (HAEC) have been studied in this work using NAA and PSi as substrates. Previously, we have reported the development of Col-coated silicon microstructures to study the effect of the topography on the behaviour of HAEC [15, 16, 42]. HAEC cell line is one of the most commonly used models in the study of the endothelial dysfunction and its capacity to adhere to the substrate and to produce cell adhesion molecules make them a good tool for screening emerging cardiovascular therapies [43].

Herein, the goal of our study is to fabricate Col- and Fn-coated NAA and macroporous PSi (MacroPSi) substrates and to study the effects of topography and coating of such substrates on endothelial cells behaviour.

## Methods

### Fabrication of macroporous silicon (MacroPSi) and nanoporous anodic alumina (NAA)

MacroPSi samples were fabricated by anodic dissolution of boron-doped p<100>silicon wafers with a resistivity of 10-20 Ω-cm in HF solution. MacroPSi substrates were prepared in a custom-made Teflon etching cell using an electrolyte of hydrofluoric acid (40%) in N, N dimethylformamide (DMF) (1:10) with a current density of 5 mA/cm^2^ for 1 h [44]. Then the samples were rinsed with pentane and dried under a nitrogen flow. Substrates with a pore diameter of 1-1.2 μm and a pore depth of 20 μm were obtained.

NAA was fabricated from high purity 99.999% aluminum foils (Goodfellow Cambridge Ltd.) using a two-step anodization process. The first anodization was performed in 0.3 M oxalic acid (H_2_C_2_O_4_) solution at 40 V/5 °C for 20 h [31, 32]. After removing porous alumina by a wet chemical etching in a mixture of 0.4 M phosphoric acid (H_3_PO_4_) and 0.2 M chromic acid (H_2_CrO_4_) at 70 °C, a second anodization was performed under the same conditions as was used in the first electrolysis. The depth and pore diameter was controlled by changing the second anodization time. NAA substrates with a pore diameter of 30-40 nm and pore depth of 50 μm approximately were obtained.

### Surface characterization

MacroPSi and NAA oxide samples were morphologically characterized by scanning electron microscopy (SEM) using an FEI Quanta 600 environmental scanning electron microscope (Hillsboro, OR, USA) operating at an accelerating voltage between 15 and 25 KeV. The roughness and topography of the substrates were measured by atomic force microscopy (AFM; Agilent Technologies,) using tapping mode in the air at room temperature.

### Surface functionalization

To improve surface compatibility of the substrates for cell culture, all the samples were modified with protein via the covalent-binding method. First, MacroPSi substrates were oxidized at 600 °C for 15 min. Then, silicon and alumina samples were treated with 30% hydrogen peroxide at 70 °C for 1 h in order to create reactive hydroxyl groups on the surface. The substrates were then washed with deionized water and dried in a gas nitrogen flow. Subsequently, the samples were reacted in an APTES (Sigma-Aldrich) by the exposure to a 10% (*v*/v) solution in anhydrous toluene for 1 h at room temperature. Then, samples were washed in succession with toluene, ethanol, and deionized water and dried under gas nitrogen flow. Afterwards, the samples were thermally cured at 110 °C overnight. The reaction with GTA was performed by exposure to a 10% (*v*/v) solution in anhydrous ethanol (Electron Microscopy Sciences) for 1 h at room temperature. The samples were rinsed with ethanol, deionized water and dried with nitrogen. Finally, the samples were incubated with Col from lyophilized bovine Achilles tendon (Sigma-Aldrich) in a 10 mg/mL solution in phosphate buffered saline (PBS) or Fn from bovine plasma (Sigma-Aldrich,) in a 0.1 mg/mL solution in PBS and stored at 4 °C overnight.

### Cell seeding and culture

HAEC were purchased from Cascade Biologics TM (Portland, USA) and at the 5th passage were thawed and seeded on Nunclon™ surface 12-well plates in the presence or absence (in the case of control conditions) of sterilized PSi and NAA substrates, at a density of approximately 4.4 × 10^4^ viable cells/mL. Throughout the experiment, cells were maintained in M200 medium supplemented with 2% (*v*/v) low serum growth supplement, 10 mg/mL gentamicin, 0.25 mg/mL amphotericin B (all from Life Technologies; Paisley, UK), 100 U/mL penicillin and 100 mg/mL of streptomycin (Labclinics, Barcelona, Spain). Cells were incubated at 37 °C in a humidified incubator (Heracell 150; Madrid, Spain) with an atmosphere containing 5% CO_2_.

### Cell viability and cytotoxicity

Cell viability was assessed by morphology using phase-contrast microscopy and by trypan blue dye exclusion test (Merck). At least a 97% of viable cells was required in order to guarantee the proper development of each set of experiments.

The extent of cytotoxicity in each experimental condition was determined by a colorimetric assay that measures lactate dehydrogenase (LDH) activity (The LDH Cytotoxicity Detection Kit; Roche Applied Science, Germany). LDH is an intracellular enzyme that is released into the extracellular media when the cellular membrane is compromised as a result of adverse conditions. In the present work, LDH activity was measured in cell-free culture supernatants collected 1, 2, 4, and 7 days after cells incubation on silicon or alumina substrates. A blank control (cells seeded in the multi-well plate in the absence of silicon or alumina surface) was used as a calibrator in all the experiments. Blank control values were set at 100% and the other conditions were calculated in relation to this reference value.

### Morphological analysis by scanning Electron microscopy (SEM)

HAEC were cultured on the functionalized silicon and alumina substrates for 2 and 7 days. After cell culture experiments, culture media were removed and cells were washed twice with PBS at 37 °C and afterwards fixed, as previously described [15]. Afterwards, HAEC adhesion to the functionalized substrates, morphology and proliferation were assessed using SEM (JEOL model JSM-6400), as described further below.

### Morphological characterization by confocal fluorescence microscopy (CFM)

HAEC were cultured on the functionalized substrates for 2 and 7 days. After cell culture experiments, culture media were removed and cells were washed twice with PBS at 37 °C and afterwards fixed, as previously described [15]. Actin-stain 670 phalloidin (Tebu-Bio) was used to stain the actin filaments of cytoskeleton (200 nM, 30 min), while NucGreen Dead 488 (Life Technologies) was used to stain the nuclei (2 drops/mL; 10 min). The fluorescence images were acquired using a Nikon Eclipse TE2000-E inverted microscope, equipped with a C1 laser confocal system (EZ-C1 software, Nikon). Six hundred and thirty-three laser and 488 nm argon laser were used as excitation sources for Phalloidin and NucGreen, respectively. Actin filaments and nuclei stain visualization using CFM were used to assess cellular morphology and adhesion, as described below.

### Cell behaviour assessment: adhesion, morphology, and proliferation

Cell adhesion to substrates was assessed by quantifying the number of cells attached to such structures. Cell morphology was defined as the combination of circularity, alignment to the substrate structures, and filopodia presence. On the one hand, circularity was calculated as the ratio between the minimum and maximum diameters. Values range from 0 to 1, where 0 represents an elongated cell and 1 a perfect circular shape. On the other hand, alignment and filopodia presence was estimated by visual assessment.

Cell proliferation was calculated as the ratio of cell number at day 7 minus cell number at day 2.

### Statistical analyses

One-way analysis of variance (ANOVA) with Bonferroni post-hoc test was used for multiple comparisons. Paired and unpaired T-tests for normal distribution were used for comparisons of two dependent or independent groups, respectively. A *p*-value < 0.05 was considered statistically significant.

A requisite for the analytical quality of the model was the control of several aspects involved in the cellular processes and analytical performance of measurements. Thus, the precision of the model was evaluated by calculating the standard deviation (SD), the standard error of the mean and the coefficients of variation (CV) of the variables. All the results were analysed with the Statistical Package for the Social Sciences (SPSS) software (version 23.0).

## Results and discussion

### Fabrication and characterization of MacroPSi and NAA substrates

**Fig. 1:**
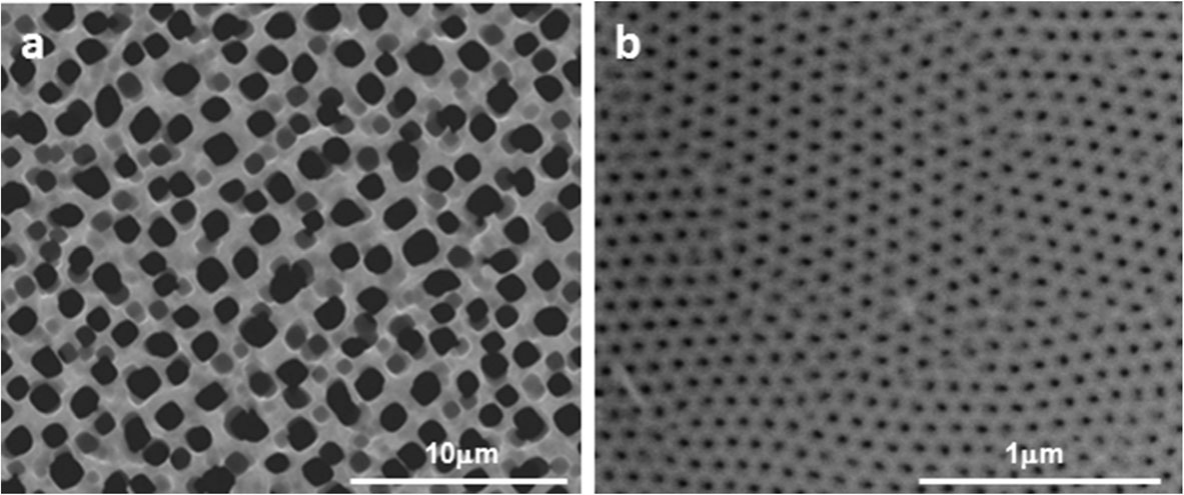
Surface topography by scanning electron microscopy. SEM images of **a** MacroPSi and **b** NAA surfaces

To study the cellular response on different porous materials and on different topography, MacroPSi substrates and NAA samples were fabricated. Figure 1 shows SEM images of the top surface morphology of these porous materials. It is represented the uniform porosity of the MacroPSi with a pore size of 1-1.2 μm. In the case of the NAA samples, the anodic oxidation of the aluminium in oxalic acid results in the pore diameters about 30-40 nm.

**Fig. 2:**
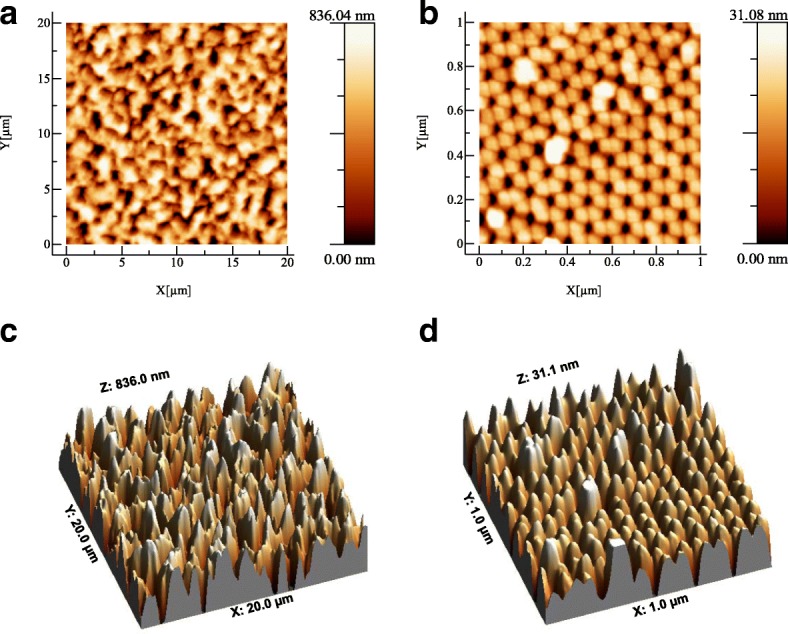
Surface topography by AFM. Two-and three-dimensional AFM images of MacroPSi (**a** and **c**) and NAA (**b** and **d**) substrates

Surface topography of the MacroPSi and NAA was determined using tapping mode AFM (Fig. 2). The corresponding roughness parameters were obtained from the images. With the increase of the pore size the mean square roughness increase, from 7.8 nm (NAA) to 0.2 μm (MacroPSi) (Fig. 2a and c). Figure 2b and d show two- and three-dimensional AFM of the second NAA substrates. The surface shows a uniform close-packed array of honeycomb structures, each containing a central pore to the substrate whose diameter is 30-40 nm.

In this work, the surfaces of the different substrates were bio-activated to promote cell adhesion and surface stability. The chemical modification of the material surfaces with amino-silanes and homobifunctional aldehydes has been shown to efficiently immobilize proteins and antibodies [40, 41]. Flat silicon used as a control in this work, MacroPSi, and NAA samples were functionalized with APTES and GTA crosslinking chemistry, which provides –NH_2_ and -CHO functional groups, respectively, to create a stable covalent binding of Col or Fn.

### Cytotoxicity of PSi and NAA

**Fig. 3:**
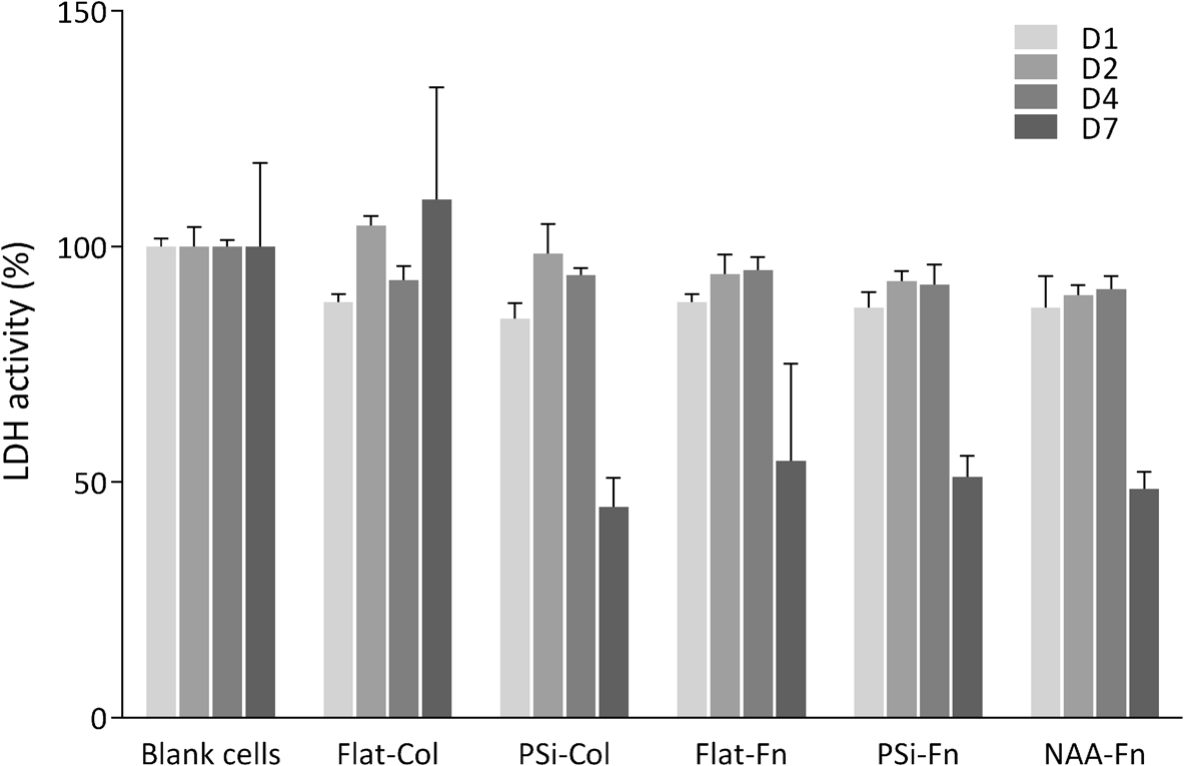
Cell cytotoxicity. Cytotoxicity observed after D1-D7 of HAEC incubation on a regular 12-well plate (blank condition) and in the presence of different substrates functionalized silicon coated with Col or Fn (Flat-Col, PSi-Col, Flat-Fn, and PSi-Fn) and alumina coated with Fn (NAA-Fn). No statistical differences were found in any condition tested. *p < 0.05 versus blank cells condition

Cytotoxicity was assessed by measuring LDH activity after 1, 2, 4, and 7 days (D1-D7) of cells incubation with flat or porous Si substrates coated with Col (Flat-Col and PSi-Col) or Fn (Flat-Fn and PSi-Fn) and alumina substrates coated with Fn (NAA-Fn). Blank control values (cells seeded in the absence of substrates) were set at 100% and the other conditions were calculated in relation to this reference value. As shown in Fig. 3, no cytotoxicity was observed at any condition, since no statistically significant changes were observed.

### Cell adhesion

**Fig. 4:**
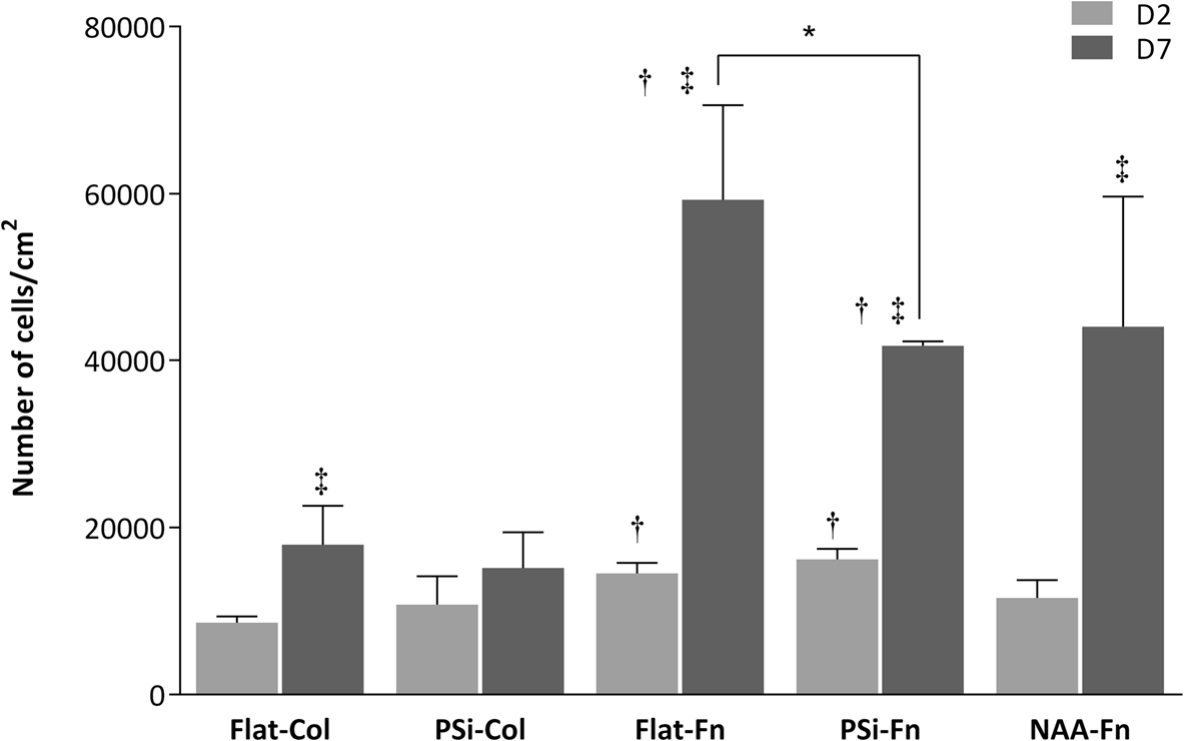
HAEC adhesion. Attachment of HAEC after D2 or D7 of culture on different substrates. *p < 0.05 between different topographies. †*p* < 0.05 versus Col-functionalized structures. ‡ *p* < 0.05 versus D2

HAEC adhesion to Col- and Fn-functionalized PSi and NAA substrates was assessed with SEM and CFM after 2 and 7 days (D2 or D7, respectively) of culture. As observed in Fig. 4, the topography of the substrates had an impact on cells adhesion in Fn-functionalized structures. In this sense, Flat-Fn had a higher number of adhered cells than PSi-Fn at D7 (*P* < 0.05), although no differences were observed at D2.

If we compare PSi-Fn and NAA-Fn in order to compare pore size, no differences were observed at D2 nor in D7. However, protein-functionalization of the surfaces had a higher impact on cells adhesion. Results showed that cells have a better predilection to adhere to Fn- than to Col-functionalized surfaces (*P* < 0.05) regardless of the topography (Flat and PSi) and the times tested (D2 and D7). In addition, cells adhesion was also affected by time culture since adhesion increased in a time-dependent manner in Flat-Col, Flat-Fn, PSi-Fn, and NAA-Fn surfaces (D2 vs D7; *P* < 0.05). These data demonstrate that cell adhesion is affected by topography as well as surfaces functionalization and culture time.

### Cell morphology

**Fig. 5:**
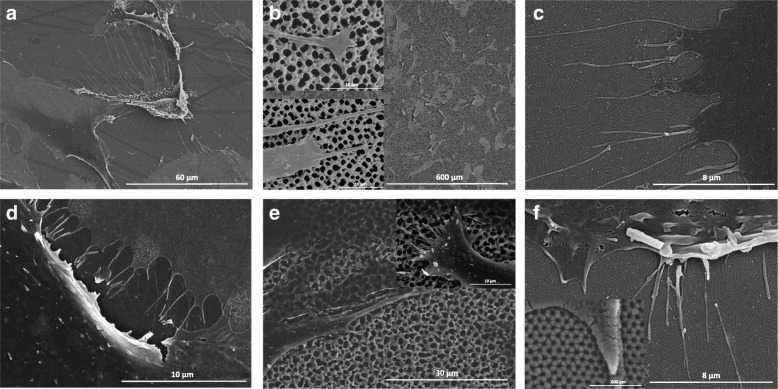
Morphological analysis of HAEC. SEM micrographs of cells at D2 on Flat-Col (**a**), PSi-Col (**b**), Flat-Fn (**d**), PSi-Fn (**e**) and, NAA-Fn surfaces (**c** and **f**)

**Fig. 6:**
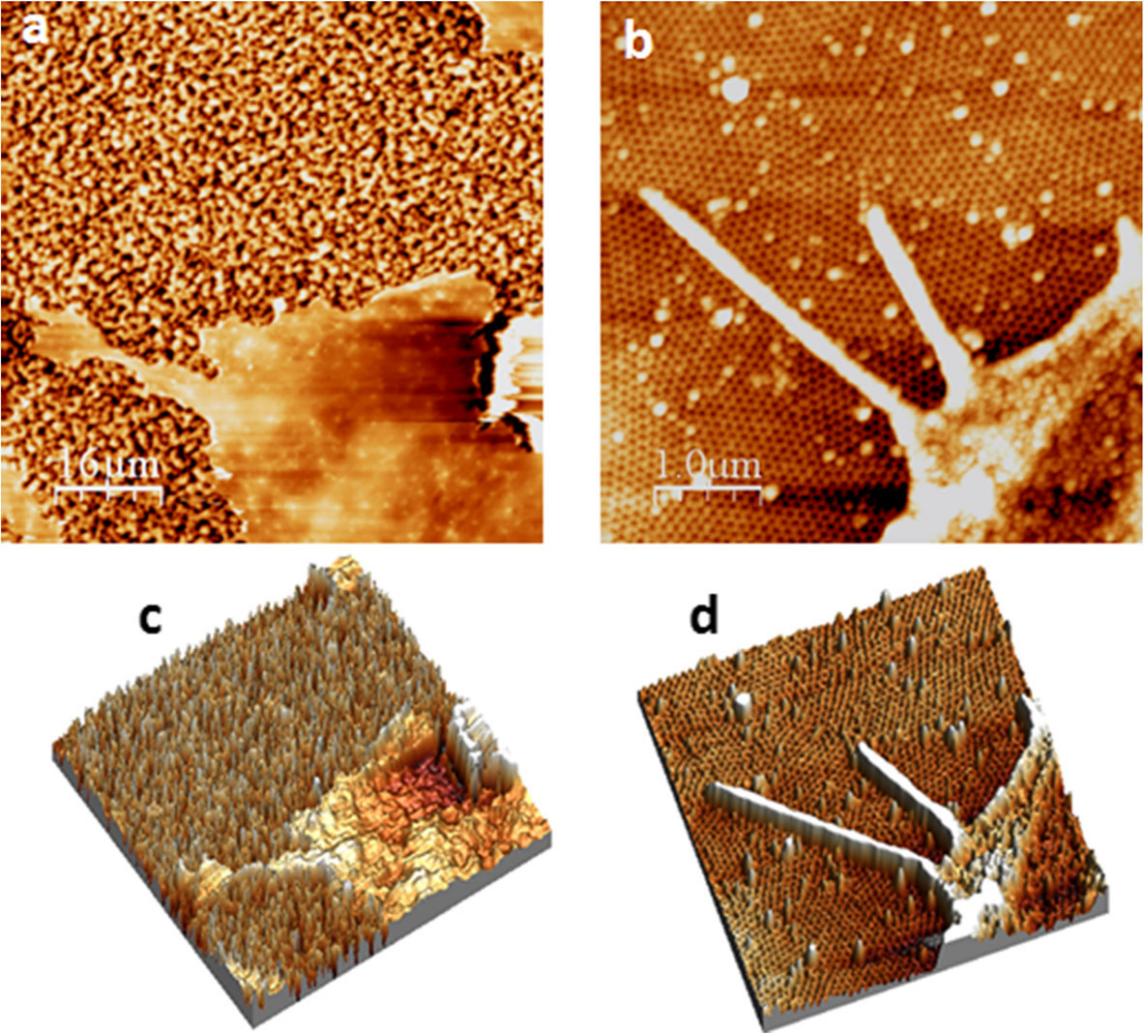
Cell spreading and filopodia formation of HAEC. AFM images of HAECs at D2. Two-dimensional images of PSi-Fn (**a**) and NAA-Fn substrates (**b**). Three-dimensional images of PSi-Fn (**c**) and NAA-Fn substrates (**d**)

Cell morphology (filopodia presence and cell circularity) is a response to topographical features of the substrate surface, and how the cells adhere and spread on the surface influences their behaviour. SEM images of HAEC at D2 of culture on PSi and NAA substrates are illustrated in Fig. 5. Cells presented flattened cell morphology irrespective of surfaces’ topography (Fig. 5a and d versus Fig. 5b and e). The cell surface is covered by microvilli and the development of the filopodia at the borders of the cell is present when the cells are cultured on NAA (Fig. 5c and f). In concordance with our previous study, cells incubated on PSi surfaces have a well-spread cytoskeleton with protrusions out of the cell membrane and, part of it penetrates into the porous [15]. Substrates’ functionalization had no impact on the presence of cell filopodia since no differences were observed in Col- (Fig. 5a and b) versus Fn-functionalized surfaces (Fig. 5d and e). We also investigated the impact of pore size using different materials, PSi and NAA, on cell spreading and filopodia formation (Fig. 6). On PSi surfaces, lamellipodia is observed while thin filopodia are present on NAA surfaces.

**Fig. 7:**
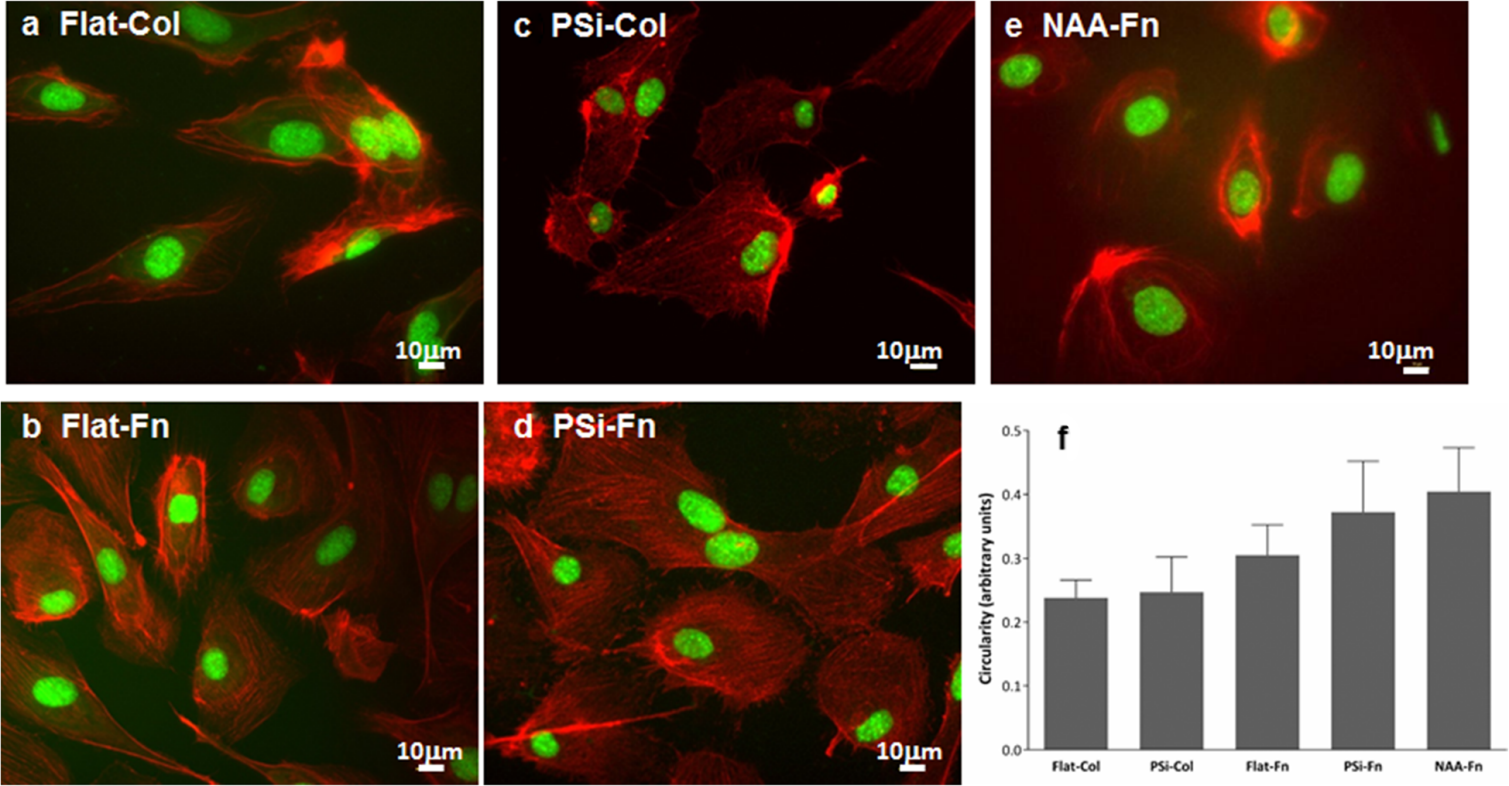
Morphological characterization of HAEC and cell circularity. Confocal fluorescence microscopy (CFM) images of HAECs at D2 on flat silicon (**a** and **b**), MacroPSi (**c** and **d**), and NAA (**e**) modified with Col and Fn. Cells were stained with NucGreen for the nucleus and Phalloidin for actin filaments. The circularity of HAEC at D2 on MacroPSi and NAA substrates Col- and Fn-functionalized (**f**)

Cell circularity was also analysed with values between 0 and 1, where 0 represents an elongated cell and 1 value represents a perfectly circular shape. As stated in Fig. 7f, no statistical changes were observed between any of the conditions tested. However, cells tend to be more circular when incubated on Fn- (Flat-Fn or PSi-Fn) versus Col-functionalized surfaces (Flat-Col or PSi-Fn). Cells incubated on NAA-Fn showed the highest circular shape. Similar results can be derived from CFM images (Fig. 7a-e).

### Cells proliferation

**Fig. 8:**
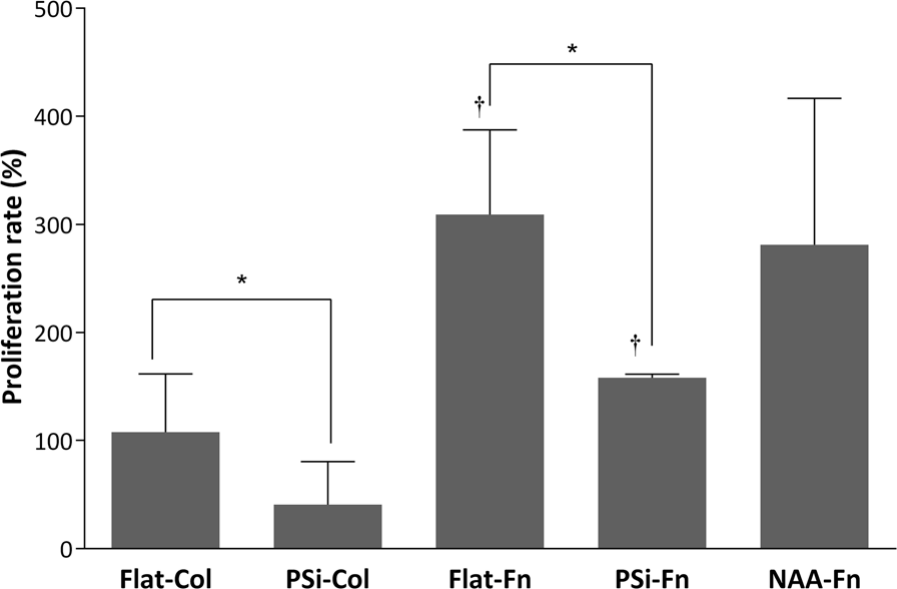
Cell proliferation. HAEC proliferation incubated for D2 and D7 on Flat, PSi and NAA substrates functionalized with Col or Fn. *p < 0.05 differences between Flat and Psi. †*p* < 0.05 versus Col

As observed in Fig. 8, cells proliferation was higher when cultured on flat (Flat-Col and Flat-Fn) surfaces than on MacroPSi (PSi-Col and PSi-Fn) surfaces (*P* < 0.05).

Surfaces functionalization also affected proliferation, since cells proliferation was higher in Fn- (Flat-Fn and PSi-Fn) than in Col-functionalized (Flat-Col and PSi-Fn) substrates (*P* < 0.05). Concerning NAA-Fn, albeit being a porous surface, cells proliferation was similar to Flat-Fn surface.

The pore size also affects the cells proliferation. Better result is obtained with NAA-Fn than on MacroPSi substrates, which suggest cells proliferation higher when the size pore is smaller.

## Conclusions

In this study, macro- and nanoporous surfaces bio-activated with Col and Fn were prepared in order to analyse the effect of the surface topography on the cell behaviour. The cell adhesion of the HAECs is affected by surface functionalization and culture time. Cells have better adhesion to Fn than Col on both flat and porous surfaces. However, substrate’s functionalization has no effect on the cell morphology. It is influenced by the pore size of the material employed. On MacroPSi lamellipodia is observed while filopodia are observed when the cells are cultured on NAA.

These results suggested that NAA and PSi can be useful culture substrates in the field of the tissue engineering because of the biocompatible nature and the ability of silicon and alumina to support cells growth.

## Abbreviations

AFM: Atomic force microscopy
ANOVA: One-way analysis of variance
APTES: 3-Aminopropyltrietoxysilane
CFM: Confocal fluorescence microscopy
Col: Collagen
CV: Coefficients of variation
DMF: N, N Dimethylformamide
Fn: Fibronectin
GTA: Glutaraldehyde
HAECs: Human aortic endothelial cells
LDH: Lactate dehydrogenase
MacroPSi: Macroporous Porous silicon
NAA: Nanoporous anodic alumina
PBS: Phosphate buffered saline
PSi: Porous silicon
SD: Standard deviation
SEM: Scanning electron microscopy
SPSS: Statistical Package for the Social Sciences
